# Federated Learning in Ocular Imaging: Current Progress and Future Direction

**DOI:** 10.3390/diagnostics12112835

**Published:** 2022-11-17

**Authors:** Truong X. Nguyen, An Ran Ran, Xiaoyan Hu, Dawei Yang, Meirui Jiang, Qi Dou, Carol Y. Cheung

**Affiliations:** 1 Department of Ophthalmology and Visual Sciences, The Chinese University of Hong Kong, Hong Kong SAR, China; 2 Department of Computer Science and Engineering, The Chinese University of Hong Kong, Hong Kong SAR, China

**Keywords:** federated learning, deep learning, ocular imaging, ophthalmology, data security, patient privacy

## Abstract

Advances in artificial intelligence deep learning (DL) have made tremendous impacts on the field of ocular imaging over the last few years. Specifically, DL has been utilised to detect and classify various ocular diseases on retinal photographs, optical coherence tomography (OCT) images, and OCT-angiography images. In order to achieve good robustness and generalisability of model performance, DL training strategies traditionally require extensive and diverse training datasets from various sites to be transferred and pooled into a “centralised location”. However, such a data transferring process could raise practical concerns related to data security and patient privacy. Federated learning (FL) is a distributed collaborative learning paradigm which enables the coordination of multiple collaborators without the need for sharing confidential data. This distributed training approach has great potential to ensure data privacy among different institutions and reduce the potential risk of data leakage from data pooling or centralisation. This review article aims to introduce the concept of FL, provide current evidence of FL in ocular imaging, and discuss potential challenges as well as future applications.

## 1. Introduction

Artificial intelligence (AI), particularly deep learning (DL), has been widely adopted in recent years to optimise the work processes in medical fields. Research and development in DL have grown significantly in capabilities and popularity in disease screening programs, automated diagnosis, treatment or prognosis prediction, and smart health care, which showed great potential to improve the clinical workflow [1,2]. In ophthalmology, DL algorithms have been developed to detect and classify various ocular diseases such as diabetic retinal diseases [3,4], age-related macular degeneration [5,6], retinopathy of prematurity [7], and glaucomatous optic neuropathy [8,9,10], using image-based data such as retinal photographs, optical coherence tomography (OCT) images and OCT angiography (OCTA) images. The advancement of DL algorithms also showed its ability in detecting and predicting systemic diseases such as diabetes [11], chronic kidney disease [12], cardiovascular events [13,14], and Alzheimer’s disease [15] based on retinal photographs. Furthermore, DL-based ocular image analysis can be incorporated with telemedicine to identify and monitor eye diseases for patients in community clinics and primary care [16].

DL is data-driven and needs to collect extensive and various training datasets to improve robustness and generalizability. Multicentre studies are becoming increasingly important in developing DL algorithms feasible in different real-world settings [17,18]. Currently, the most common paradigm for such collaborative multicentre projects is referred to as “centralised learning”, in which data from different sites is transferred and pooled into a centralised location in accordance with inter-site agreements. However, big data collection and resource sharing could raise practical concerns, and it often takes time to resolve ethical and privacy-related issues. In medical imaging, even anonymous raw images contain patients’ private information. For instance, retinal images are unique as fingerprints [19] and highly sensitive, as age [20], sex [21], cardiovascular risk factors [13], or mortality risk [22] could be predicted from fundus photographs or OCT scans. The human faces can be reconstructed from de-identified magnetic resonance imaging (MRI) scans [23]. 

Hence, to ensure data privacy and reduce the potential risk of raw data leakage in the conventional paradigm (i.e., centralised learning), the “distributed learning” paradigm [24] has been developed to distribute data across different institutions rather than combine it into a single pool. A recent advancement in distributed learning is federated learning (FL) [25,26,27], which allows multiple medical institutions to collaboratively train AI models without data sharing. It significantly facilitates AI research and development in the healthcare domain, in which data is highly valuable, and it typically needs to involve multiple centres and access to large-scale data. 

This review article aims to introduce the basic concept of FL and discuss its advantages and applications in healthcare, especially in ophthalmology, as well as its future development.

## 2. What Is Federated Learning?

**Figure 1:**
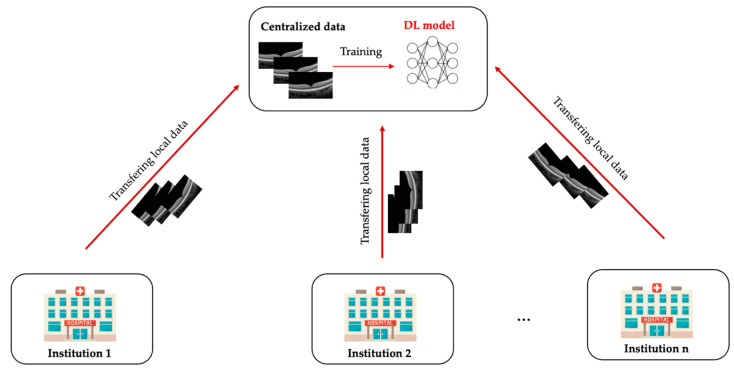
Conventional centralised learning. All participating institutions transfer their dataset to a centralised location, where the deep learning model is developed.

**Figure 2:**
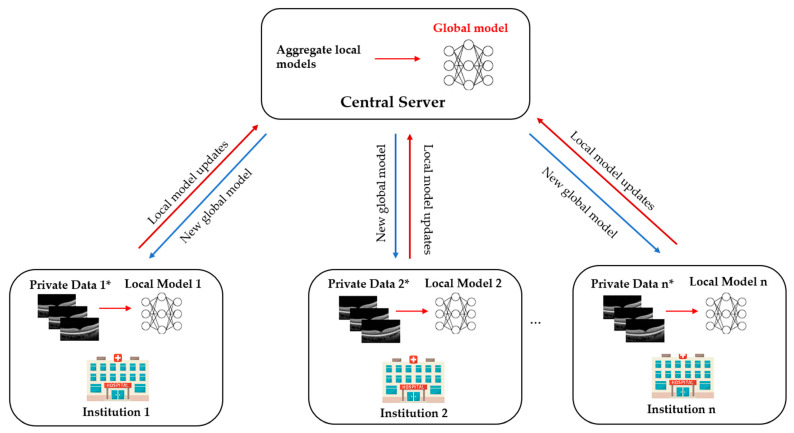
The architecture of federated learning. Each institution trains a local model on its own training dataset. All local models’ parameters are then transferred to the central server after one training epoch. The central server accumulates and aggregates all local parameters and updates the global model securely. Afterwards, the model is updated, and the aggregated parameters are redistributed to each centre for a new round of training. This process is iterated until the global model converges. (*) Optical coherence tomography (OCT) images are illustrated as private data from participating institutions. Other different ophthalmic imaging modalities (e.g., slit-lamp images, fundus photographs, OCT-angiography images) can also be used when exploring the federated learning approach in the field of ocular imaging.

Traditionally, the DL approach requires pooling all available data from multiple institutions into a central source for model training and testing (Figure 1). FL, on the contrary, is a distributed learning paradigm where multiple collaborators train a model on their own data locally and then send their model updates to a central server to be aggregated into a consensus model [28]. It avoids the need to put all the collected data in one place or directly access the sensitive data across collaborators. Each institution keeps its data locally and will not transfer or directly access data across institutions (Figure 2). The FL paradigm for model training is based on three main steps [29]: (i) initially, the global model is initialised by the central server and then distributed to each contributing institution; (ii) each institution trains the mode using its local data, and then sends the local model back to the central server; (iii) the central server aggregates all local models to update a new global model and redistributes it to all collaborators (Figure 2). These steps are repeated back and forth until the global model reaches a stable performance. The model training procedure in traditional DL and FL is the same. However, the only difference between DL and FL training paradigm is that the DL requires a chief institution to train the model on all data, while FL allows each institution to perform training locally. As only the model characteristics (e.g., model parameters or gradients) are to be sent out from institutions, this distributed training approach has great potential to ensure data privacy among different institutions and reduce the potential risk of data leakage from data centralisation. Meanwhile, it can enable the model to be trained and validated across multiple datasets to improve its robustness and generalizability. As a result, FL offers tremendous advantages in data privacy over conventional centralised learning approaches, especially in AI research and the field of healthcare.

## 3. Types of Federated Learning

**Figure 3:**
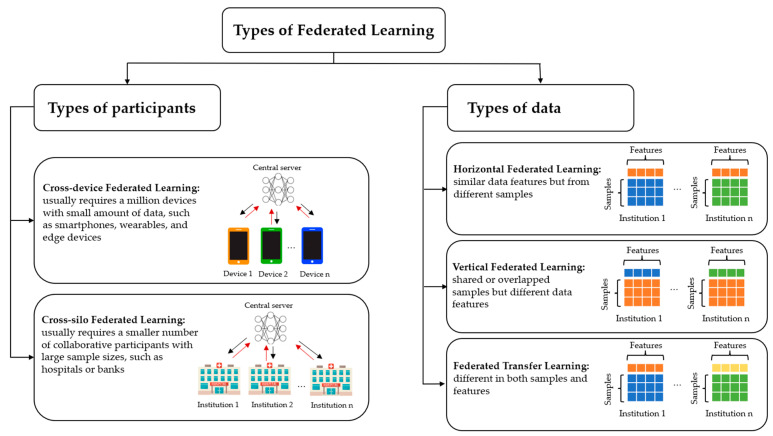
Types of federated learning.

Generally, there are two major categories of FL proposed by previous studies, focusing on the type of participants and data (Figure 3).

Based on the properties of participants (or called clients) in FL, it can be grouped into two main types, (1) cross-silo FL and (2) cross-device FL [29]. On cross-device FL, its implementation ensures that learning takes place remotely and updates a central model via a federated system. The cross-device FL usually requires a million devices (e.g., smartphones, wearables, and edge devices) with a small amount of data to participate in the training process. On the other hand, cross-silo FL allows a smaller number of collaborative participants with large sample sizes, typically reliable companies or organisations such as hospitals or banks. Moreover, in cross-device FL, the participants are not always available (e.g., poor network connection and battery status), making the participants inconsistent for each round. Generally, cross-silo FL has a much better performance consistency since they use dedicated hardware and efficient networks [30]. 

Based on the data distribution between different functions and sample spaces, FL can be categorised into horizontal FL (HFL), vertical FL (VFL), and federated transfer learning (FTL) [25,29]. HFL refers to the sample-based FL, which is introduced when data sets share similar data features but from different samples [29]. For instance, two different eye hospitals treat patients with primary open-angle glaucoma. Both hospitals may have patients with similar disease features, i.e., glaucomatous optic neuropathy, while the patients’ demographic characteristics are primarily diverse, as both hospitals are located in different places. On the other hand, VFL is utilised when there are shared or overlapped samples but differ in data features [29]. For instance, the pharmacy and radiology departments in the same hospital are two different departments with distinct features. However, both departments may have information from the same group of patients. FTL is primarily used in scenarios where datasets vary in both samples and features [25]. For instance, various institutions may be located in different regions, and based on these restrictions, users of these institutions have a few intersections. The purpose of FTL is to develop effective application-specific models in situations where data is scarce. FTL can be offered to bring about solutions for the whole sample and feature space to bridge the gap between heterogeneous datasets. 

## 4. Federated Learning Applications in Healthcare

### 4.1. Electronic Health Records

Electronic health records (EHRs) are recorded as part of routine care in most healthcare institutions, which contain patients’ medical information, including demographic information, laboratory results, medical imaging, diagnoses, treatments, and prescriptions. The primary benefits of EHRs data are improving the ease of access to patients’ health information and monitoring patients. With the advancement of AI, analysing electronic health data using DL techniques can significantly improve the decision-making process, risk assessment and disease progression, thus increasing healthcare quality. With FL technology to guarantee patient privacy, Deist et al. [31] achieved an improvement in predicting post-treatment two-year survival on more than 20,000 non-small cell lung cancer patients across eight healthcare institutes in 5 countries. Another FL framework on EHRs data proposed by Sharma et al. [32] to predict in-hospital mortality for patients in intensive care units achieved comparable performance to those trained in a centralised manner. Furthermore, within the scope of coronavirus disease 2019 (COVID-19), FL has been shown to predict acute kidney injury in patients with COVID-19 within 3 to 7 days after admission utilising EHRs data, including demographics, medical history, laboratory data, and even vital signs data [33]. Using a similar form of FL, Vaid et al. predicted mortality within seven days after hospitalisation of patients due to COVID-19 [34] by gathering EHRs data from different hospitals. Their results showed notable improvement of the federated model compared to the locally trained model and non-inferior to centralised learning. FL is becoming a promising approach for institutions that wish to collaborate with others in data-driven research utilising EHRs.

### 4.2. Internet of Things in Healthcare

The advancement of internet of things (IoT) in healthcare offers people the opportunity to monitor their health status and receive early warnings of health issues or existing conditions [35]. FedHealth, the first FTL framework for wearable healthcare, performs data aggregation through FL and builds relatively personalised models by transfer learning [36]. The framework has been achieved accurately in the auxiliary diagnosis of Parkinson’s disease and is promised to be deployed in other healthcare applications, such as elderly care, fall detection, and cognitive disease detection. Furthermore, a recent study by Brophy et al. [37] utilised a FL framework for developing a model to measure continuous arterial blood pressure (ABP) using a single optical photoplethysmogram sensor without compromising the ABP accuracy and patient privacy. This non-invasively method of monitoring ABP could benefit for people suffering from cardiovascular diseases. By using the distributed learning framework, it enables multiple remote devices to train collaboratively without data sharing. The results showed equal performance between federated and non-federated frameworks, which opens up new opportunities for applying FL in wearable devices to monitor patients’ cardiovascular status remotely and accurately.

### 4.3. Medical Image Analysis

FL is now being applied in a wide range of applications in medical image analysis. This distributed learning approach has the potential to develop a robust model which leverages large and multiple diverse medical image datasets obtained from different institutions, while ensuring patient privacy and data ownership. Li et al. implemented and evaluated a FL system for brain tumour segmentation on MRI scans from the Brain Tumour Segmentation (BraTS) dataset [38]. The proposed FL model can achieve a comparable segmentation performance to the data-centralised training model. Another study using functional MRI data in Autism Brain Imaging Data Exchange (ABIDE) project demonstrated that FL could utilise the multi-site data to boost the neuroimage analysis performance for identifying neurological biomarkers [39]. Lee et al. (15) evaluated the feasibility and performance of FL for thyroid tumour identification from 8457 ultrasound images collected from 6 institutions. The results demonstrated that the performance of FL at each institution was comparable to that of conventional DL using pooled data, with the area under the curve of receiver operating characteristic (AUROC) from 78.88% to 87.56%. Shiri et al. [40] built a federated DL-based model for positron emission tomography (PET) image segmentation by utilising 405 PET images of head and neck cancer patients from 9 different centres. The developed FL model achieved comparable quantitative performance with respect to the centralised DL model while considering the privacy concerns and the legal and ethical problems in medical data sharing in clinical institutions.

FL has been used for medical image analysis to detect COVID-19 lung abnormalities from chest X-rays and CT-scans images [41,42,43]. FL was used to train a DL model using inputs of vital signs, laboratory data, and chest X-rays from 20 institutions in different countries [43]. FL allowed the model to train faster amid the ongoing pandemic and generalise the heterogeneous, unharmonised datasets for predicting clinical outcomes in COVID-19 patients. Dou et al. [41] demonstrated a FL method to build a deep convolutional neural network-based AI model for automated detection of lesions from COVID-19 CT images, which performed well on external data. The result indicated the potential of FL to develop generalisable, low-cost, and scalable AI tools for image-based disease diagnosis and management, both for research and clinical care.

Furthermore, FL has shown its feasibility and effectiveness for weakly supervised classification of carcinoma in histopathology and survival prediction by using thousands of gigapixel whole slide images from multiple institutions [44]. The results demonstrated that FL could effectively assist clinicians in classifying subtypes of renal cell carcinoma and breast invasive carcinoma and address the challenges associated with the lack of detailed annotations in most real-world datasets. FL framework, therefore, has the clear potential to be applied in rare diseases where datasets are limited or in countries that lack access to pathology and laboratory services.

## 5. Current FL Applications in Ophthalmology

FL has already shown its potential in ophthalmology for different retinal diseases detection from ocular images such as OCT, OCTA, and retinal photographs.

### 5.1. Diabetic Retinopathy

Yu et al. [45] utilised the FL framework for referable diabetic retinopathy (RDR) classification using OCT and OCTA from two different institutions. The performance of the FL model was compared with the model trained with data acquired from the same institution and from another institution. The results were comparable to those trained on local data and outperformed those trained on other institutes’ data. This study demonstrated the potential for FL to be applied for the classification of DR and facilitate collaboration between different institutions in the real world.

Furthermore, the study also investigated the FL approach to apply microvasculature segmentation to multiple datasets in a simulated environment. The study designed a robust FL framework for microvasculature segmentation in OCTA images. The image datasets were acquired from four different OCT devices. The FL framework in this experiment achieved performance comparable to the internal model and the model trained with combined datasets, showing that FL can be used to improve generalizability by including diverse data from different OCT devices.

However, regardless of the promising performance of the FL approach, it is essential to consider the potential application scenario. Using OCTA images for the classification of RDR could not be feasible in the DR screening programme in the real world. Retinal fundus photography is a widely acceptable imaging modality for identifying RDR, whereas OCTA images would be helpful to detect diabetic macular ischemia. In addition, although the source of images used for microvasculature segmentation was obtained from different OCT devices, the sample size of the datasets was small. Therefore, sample size justification would be needed to make the result of the FL approach more meaningful.

### 5.2. Retinopathy of Prematurity

Retinopathy of prematurity (ROP), a leading cause of childhood blindness worldwide, is a condition characterised by the growth of abnormal fibrovascular retinal structures in preterm infants. Hanif et al. [46] and Lu et al. [47] explored the FL approach for developing a DL model for ROP. Lu et al. [47] utilised, trained, and validated a model on 5245 ROP retinal photographs from 1686 eyes of 867 premature infants in neonatal intensive care of seven hospital centres in the United States. The images were labelled with clinical diagnoses of plus disease (plus, pre plus, or no plus) and a reference standard diagnosis (RSD) by three image-based ROP graders and the clinical diagnosis. In most DL model comparisons, the models trained via the FL approach achieved a performance comparable with those trained via the centralised learning approach, with the AUROC ranging from 0.93 to 0.96. In addition, the FL model performed better than the locally trained model using only a single-institution data set in 4 of 7 sites in terms of AUROC. Moreover, the FL model in this study maintained its consistency and accuracy to heterogenous clinical data sets among different institutions, which varied in sample sizes, disease prevalence and patient demographics.

In the second experiment, Hanif et al. [46] demonstrated the potential ability of FL to harmonise the difference in clinical diagnoses of ROP severity between institutions. Instead of using the consensus RSD, a FL model was developed based on ROP vascular severity score (VSS). In this study, there was a significant difference in the level of VSS in eyes with no plus disease. VSS could be subjective, with considerable variation between experts in clinical settings that may affect clinical or epidemiology research [48]. However, according to this study, the FL model could standardise the difference in clinical diagnoses across institutions without centralised data collection and consensus of experts. Based on the results of this study, the FL model provides a generalisable approach to assessing clinical diagnostic paradigms and disease severity for epidemiologic evaluation without sharing patient information.

These two studies demonstrated the utility of FL framework in ROP, allowing collaboration between different institutions while protecting data privacy. However, these studies were still conducted under a simulated environment. Practical issues during clinical implementation such as communication efficiency or bias of data among participating centres could not be identified in these studies. Such challenges will be further discussed in the section below.

## 6. Challenges and Vulnerabilities

Although FL-based models show promising performance and tremendous potential in ophthalmology, most of these models were developed in a simulated environment and without proper testing on unseen datasets. There are still unsolved issues for applying FL in real-time and real-world clinics. 

### 6.1. Data Heterogeneity

Medical data from different institutions are highly heterogeneous. Different institutions in FL have varying amounts of data with different properties such as vendors, imaging protocol and patient population or demographic. These data heterogeneity in FL are usually known as non-independent and identically distributed (non-IID) [49,50]. For example, in the diagnosis of diabetic macular oedema, OCT images collected from different institutions have uniformly distributed labels. However, the image appearance can vary greatly due to different imaging protocols and OCT machines used in hospitals, e.g., different intensity and contrast. Data heterogeneity from participating institutions will result in weight divergence of the local model, which represents the difference between weights updated based on non-IID multi-modal data and centralised data [51], and further deteriorate significantly the performance FL model [50]. The most popular FL algorithm is Federated Averaging (FedAvg), which was demonstrated to be able to handle heterogeneous data. However, FedAvg does not perform well on highly skewed non-IID data and may require much more communication rounds to converge [49]. Zhao et al. reported that the accuracy of FL reduces significantly up to ~55% for neural networks trained on highly skewed non-IID data [50]. Although a large number of approaches have been proposed for handling non-IID data in FL, including data sharing [50], knowledge distillation [52], and personalised FL [53,54], it remains challenges related to data privacy or communication cost and mainly focuses on HFL scenarios [55]. Therefore, further technical studies are needed to find out effective methods to tackle non-IID in FL.

### 6.2. Bias

Bias is an issue that a model tends to predict a certain kind of outcome more than others due to the imbalance of training datasets (e.g., insufficient or no data for specific diseases or subpopulations) [56]. For example, a binary classification task to determine whether referable or non-referable DR uses a dataset containing fundus photographs. If the input classes in the training data are imbalanced (e.g., more non-referable DR than referable DR), the final model will be biased toward the over-represented class [57]. The problem of bias could be aggravated in FL systems because each participant will contribute their own bias to the global server and may even generate new ones. In addition to bias in the training data, the FL systems could induce bias due to the variety of devices, the difference in network bandwidth and latency or compute performance [58]. Several recent studies have further proposed methods to mitigate the bias in FL based on the degree of bias from each FL participant affecting the global server [59,60,61,62,63]. After estimating the level of bias, this information is introduced to the global server to modify the algorithm and further aggregate participants’ updates. However, these approaches might not be feasible as they require additional information from client data distribution which may leak sensitive information [64]. That is why future work is needed to identify the approaches to mitigate bias in FL.

### 6.3. Privacy and Security

Although FL has been proven to be effective in improving the privacy of patients by keeping data locally, there remain some privacy-related challenges associated with FL that require attention. During the training process or the interaction between participants and the central server, adversaries may reveal sensitive information and reconstitute the patients’ data by sharing model updates. It has been shown that even a tiny portion of intermediate results, such as gradient information, can be attacked, resulting in reconstruction and interference of original data [65]. Furthermore, due to the fact that FL is built on a large number of participants (especially cross-device FL), malicious users may be able to generate false outputs to manipulate the DL model. In order to overcome these issues, there are privacy-preserving technologies that can be used to enhance FL’s privacy by utilising secure multi-party computation [66], homomorphic encryption [67] or differential privacy [68]. Although these methods improve the privacy of model updates or prevent poisoning attacks from malicious users, they may reduce model performance or system efficiency. This requires the researchers to make efforts on the trade-off between privacy protection and model performance and provide personalised privacy protection.

### 6.4. Communication Cost

Communication has been considered a critical bottleneck in FL. Federated networks, including many devices (e.g., millions of desktops) and transmission via the network may become slower than local transmission, especially when the local models are uploaded to the server [69]. In addition, the constant communication between participants and the global server requires a reliable network bandwidth to maintain a large amount of download and upload processes. In recent years, several federated optimisation algorithms have been proposed to alleviate the communication cost in FL. Potential methods to improve the communication-efficiency is reducing the total number of communication roads [49] and reducing the size of the uploaded parameters [26,70,71]. However, compression of the model updates presents the communication-precision trade-off as it requires a large compression ratio [72]. Moreover, these methods may also negatively affect the model performance in handling the heterogeneity of decentralised data [72]. In addition to improving the communication between the local and central server, each participating in federated networks need to prepare strong computing resources (e.g., graphics processing unit) and robust network connections between different clinics for data pre-processing. 

## 7. Future Directions

Ophthalmology is a medical speciality driven by imaging that has unique opportunities for implementing DL systems. Ocular imaging is not only fast and cheap compared to other imaging modalities such as CT or MRI scans but contains essential information on ocular and systemic diseases. Utilising FL in prior DR and ROP studies illustrates the potential ability to overcome privacy challenges and inspires further deployment of FL in other ophthalmic diseases. In the future, FL applications and developments in real-world clinics are warranted.

### 7.1. Multi-Modal Federated Learning

In ophthalmology, there are diverse data from different modalities such as fundus photography, OCT, OCTA, and visual field (VF) with different protocols. With such a wide range of modalities, using one modality alone is often insufficient to detect alterations and diagnose diseases. Glaucoma, for instance, is diagnosed based on a combination of intraocular pressure measurement, colour fundus photograph, VF examinations and peripapillary retinal nerve fibre layer (RNFL) thickness evaluation. A DL algorithm developed based on RNFL thickness without referring to the VF, or relevant clinical diagnostic data may not be enough to diagnose glaucoma in real-world setting. Recently, Xiong et al. trained and validated a bimodal DL algorithm to detect glaucomatous optic neuropathy (GON) from both OCT images and VF [73]. The diagnostic performance of the proposed DL algorithm reached an AUROC of 0.950 and outperformed 2 single modals trained by only VF or OCT data (AUROC, 0.868 and 0.809, respectively). In addition, the model achieved comparable performance to experienced glaucoma specialists, suggesting that this multi-modal DL system could be valuable in detecting GON.

Apart from glaucoma, OCT and OCTA have become necessary non-invasive imaging modalities for quantitative and qualitative assessment of retinal features (e.g., retinal thickness and retinal fluid) in many retinal diseases such as AMD and DR. A recent study by Jin et al. demonstrated the efficacy of a multimodal DL model using OCT and OCTA images for the assessment of choroidal neovascularisation in neovascular AMD, which achieved comparable performance to retinal specialists with an accuracy of 95.5% and an AUROC of 0.979 [74]. In addition to ocular imaging data, EHRs also contain various information, including past medical history and systemic features. Incorporating EHRs data offers an outstanding opportunity to better understand complex relationships between systemic and ocular diseases. Data from medical history or laboratory, such as blood pressure and glycated haemoglobin, can be used to improve the predictive power of AI systems. Therefore, it is necessary to build and implement FL systems to support multi-modal data from different modalities to enhance the performance of DL in early detection and disease management. Several existing studies proposed a multi-modal FL framework using data modalities showing promising results. Recently, Zhao et al. have proposed a multi-modal framework that enables FL systems to work better with collaborators with local data from different modalities and clients with varying setups of devices compared to a single modality [75]. Another study by Qayyum et al. suggested a framework using clustered FL-based methods for an automatic diagnosis of COVID-19 that would allow remote hospitals to utilise multi-modal data, including chest X-rays and ultrasound images [76]. Additionally, the clustered FL presented a better performance in handling the divergence of data distribution compared to conventional FL.

### 7.2. Federated Learning and Rare Ocular Diseases

In addition, FL is expected to help in the future in diagnosing, predicting, and treating rare or geographically uncommon diseases such as ocular tumours or inherited retinal diseases, where currently there are challenges due to low incidence rates and small datasets [77,78]. Connecting multiple institutions on a global scale could improve clinical decisions regardless of patients’ location and demographic environment. Fujinami-Yokokawa Y et al. [79] trained and validated a DL system for automated classification among ABCA4-, EYS-, and RP1L1-associated retinal dystrophies using a Japanese Eye Genetics Consortium dataset of 417 images (fundus photographs and FAF images). Although the DL system could provide an accurate diagnosis of three inherited retinal diseases, there is limited phenotypic heterogeneity within each group, and the dataset is from a specific ethnic population. Recently, FL has shown its feasibility and effectiveness for weakly supervised classification of carcinoma in histopathology and survival prediction by using thousands of gigapixel whole slide images from multiple institutions [44]. The study demonstrated the potential of the FL framework to be applied in rare diseases where datasets are limited or in countries that lack access to pathology and laboratory services. Therefore, FL is a promising approach for greater international collaboration to develop valuable and robust DL algorithms for rare ocular diseases.

### 7.3. Blockchain-Based Federated Learning

The development of FL could further combine with the next generation of technology, potentially blockchain technology, to improve the privacy mechanism. Blockchain is a decentralised ledger innovation predicated on privacy, openness, and immutability, which has been used in the healthcare system to manage genetic information and EHRs [80,81]. Blockchain network has also been applied in ophthalmology to detect myopic macular degeneration and high myopia using retinal photographs from diverse multi-ethnic cohorts in different countries [82]. The study suggested that adopting blockchain technology could increase the validity and transparency of DL algorithms in medicine. With its immutability and traceability, blockchain can be an effective tool to prevent malicious attacks in FL. The immediate model updates, either local weights or gradients, can be chained in a cryptographical way offered by blockchain technology to maintain their integrity and confidentiality. Thus, integrating FL and blockchain could effectively allow the processing of vast amounts of data created practically in healthcare settings and improve data security and privacy by offering security and effective points for the deployment of the model [83].

### 7.4. Decentralised Federated Learning

In FL system, a central server usually orchestrates the learning process and updates the model upon the training results from clients. However, such star-shaped server-client architecture decreases the fault tolerance, does not solve the problem of information governance, and requires a powerful central server, which may not always be available in many real-life scenarios with a very large number of clients [84,85]. Thus, the fully decentralised FL that replaces the communication between central server and each client by interconnected clients’ peer-to-peer communication was proposed to address the above-mentioned problems. Recently, swarm learning, a decentralised learning system without central server, was introduced to build the models independently on private data at each individual site and support data sovereignty, security, and confidentiality by utilising edge computing, blockchain-based peer-to-peer networking and coordinator [84]. Saldanha et al. proved that swarm learning can not only be used to detect COVID-19, tuberculosis, leukaemia and lung pathologies but also to predict clinical biomarkers in solid tumours and yield high-performing models for pathology-based prediction of BRAF mutation and microsatellite instability (MSI) status [86]. 

### 7.5. Federated Learning and Fifth Generation (5G) and Beyond Technology

With the advent of wireless communications over the past few decades, the recent 5G and beyond technology has already been launched and provided low latency, high transmission rate, and high reliability compared to existing networks [87]. An efficient 5G network could address the issue of communication latency and network bandwidth in the FL framework. Moreover, the 5G network has been implemented in managing COVID-19 patients by video telemedicine in real-time [88]. In the field of ophthalmology, 5G technology has been applied in ophthalmology to conduct real-time tele-retinal laser photocoagulation for the treatment of DR [89]. This evidence suggests the potential of integration FL and 5G technology to allow pre-processing, training, and processing data in real-time.

## 8. Conclusions

FL creates a reliable and collaborative DL model for multi-institution collaborations without compromising the privacy of data, which will be critical in ophthalmology healthcare, especially in ocular image analysis. More research is warranted in the field of ophthalmology to investigate how to apply FL efficiently and effectively in real-time and real-world clinical settings.
